# Racial and Socioeconomic Disparities in CKD in the Context of Universal Health Care Provided by the Military Health System

**DOI:** 10.1016/j.xkme.2021.08.015

**Published:** 2021-10-23

**Authors:** Jenna M. Norton, Lindsay Grunwald, Amanda Banaag, Cara Olsen, Andrew S. Narva, Eric Marks, Tracey P. Koehlmoos

**Affiliations:** 1 Department of Preventive Medicine and Biostatistics, Uniformed Services University of the Health Sciences, Bethesda, MD; 2 Department of Medicine, Division of Nephrology, Uniformed Services University of the Health Sciences, Bethesda, MD; 3 Henry M. Jackson Foundation for the Advancement of Military Medicine, Bethesda, MD; 4 College of Agriculture, Urban Sustainability & Environmental Sciences, University of the District of Columbia, Washington, DC

**Keywords:** Chronic kidney disease, disparities, health care coverage, health insurance, race, social determinants of health, socioeconomic status

## Abstract

**Rationale & Objective:**

Health-impeding social determinants of health—including reduced access to care—contribute to racial and socioeconomic disparities in chronic kidney disease (CKD). The Military Health System (MHS) provides an opportunity to assess a large, diverse population for CKD disparities in the context of universal health care.

**Study Design:**

Cross-sectional study.

**Setting & Participants:**

MHS beneficiaries aged 18 to 64 years receiving care between October 1, 2015, and September 30, 2018.

**Predictors:**

Race, sponsor’s rank (a proxy for socioeconomic status and social class), median household income by sponsor’s zip code, and marital status.

**Outcome:**

CKD prevalence, defined by *International Classification of Diseases, Tenth Revision* codes and/or a validated, laboratory value-based electronic phenotype.

**Analytical Approach:**

Multivariable logistic regression compared CKD prevalence by predictors, controlling separately for confounders (age, sex, active-duty status, sponsor’s service branch, and depression) and mediators (hypertension, diabetes, HIV, and body mass index).

**Results:**

Of 3,330,893 beneficiaries, 105,504 (3.2%) had CKD. In confounder-adjusted models, the CKD prevalence was higher in Black versus White beneficiaries (OR, 1.67; 95% CI, 1.64-1.70), but lower in single versus married beneficiaries (OR, 0.77; 95% CI, 0.76-0.79). The prevalence of CKD was increased among those with a lower military rank and among those with a lower median household income in a nearly dose-response fashion (*P* < 0.0001). Associations were attenuated when further adjusting for suspected mediators.

**Limitations:**

The cross-sectional design prevents causal inferences. We may have underestimated the CKD prevalence, given a lack of data for laboratory tests conducted outside the MHS and the use of a specific CKD definition. The transient nature of the MHS population may limit the accuracy of zip code–level median household income data.

**Conclusions:**

Racial and socioeconomic CKD disparities exist in the MHS despite universal health care coverage. The existence of CKD disparities by rank and median household income suggests that social risks may contribute to both racial and socioeconomic disparities despite access to universal health care coverage.

## Graphical abstract

**Figure:**
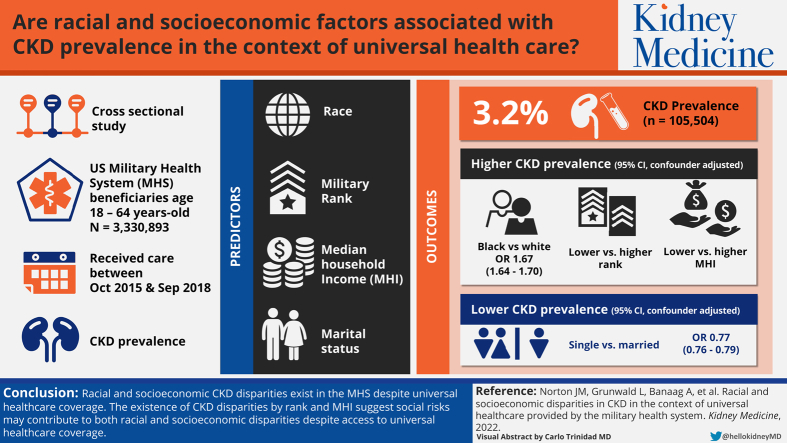

**Plain-Language Summary**

Social risk factors, such as reduced access to care, lead to disparities in chronic kidney disease (CKD) across race and class. We assessed CKD and its association with social risk factors in the Military Health System (MHS) to determine if disparities exist in the context of the universal health care provided in the MHS. About 3% of MHS beneficiaries had CKD. CKD was more common in Black than White beneficiaries, in people of lower compared to higher rank, and in people living in lower-income compared to higher-income areas. CKD was less common in single than married beneficiaries. Racial and socioeconomic CKD disparities exist in the MHS despite universal health care coverage.

Editorial, 100400

Chronic kidney disease (CKD) is common and burdensome, often leading to kidney failure and dialysis. Health-impeding social determinants of health—or social risks—have strong and well-documented associations with CKD incidence, prevalence, and progression, as well as with the substantial racial and socioeconomic disparities that characterize the disease.^1^^,^^2^ Social risks are fueled by poverty and combine and interact with clinical and biological factors to generate poor health outcomes, including CKD. They act by affecting a person’s likelihood of exposure to disease-causing agents, ability to participate in healthful behaviors, exposure to stressors and the resulting level of stress, and capacity for coping with stressors.^1^^,^^2^

Inadequate access to health care is an important social risk in the general US population, particularly among low-income individuals. In 2017, 7.4% of the total US population—and 16.2% of those living below the federal poverty line—delayed or missed necessary medical care due to cost.^3^ Uninsurance and underinsurance, common proxies for inadequate health care access, are associated with poor outcomes in CKD.^4^^,^^5^

Universal health care coverage appears to mitigate racial and socioeconomic disparities across numerous health conditions.^6^^,^^7^ Racial disparities in CKD often persist despite universal access to care,^7^^,^^8^ and have been attributed to Black-White differences in the presence of high-risk *APOL1* variants.^8^ However, socioeconomic disparities in CKD—which cannot be explained by genetic differences—are also apparent in settings with universal health care coverage, including the United Kingdom, Denmark, and Australia.^9^, ^10^, ^11^ To better understand the role of health care access in racial and socioeconomic disparities in CKD, this analysis explores the extent to which socioeconomic factors and race are associated with CKD prevalence in the context of the universal health care coverage provided within the Military Health System (MHS). We hypothesized that CKD prevalence would be elevated in beneficiaries of Black versus White race, lower versus higher rank (a proxy for socioeconomic status and social class), lower-income versus higher-income areas, and unmarried versus married status.

## Methods

### Data Source

This analysis employed data from the MHS Data Repository (MDR) via the Comparative Effectiveness and Provider Induced Demand Collaboration (EPIC) project.^12^ The MDR includes data for all in- and outpatient visits for the approximately 9.5 million MHS beneficiaries who receive care paid for by the Military’s TRICARE Health Plan. Data are available for both “direct care” interactions that occur in a Department of Defense military treatment facility and for “purchased care” interactions that occur in a civilian facility when the Military’s TRICARE Health Plan is the primary payer. Data from direct care visits include vital signs, body mass index (BMI), self-reported tobacco use, medications, and laboratory results, among other variables.^13^ However, data from purchased care visits are limited to claims data for billing and do not include outcomes or results of the clinical encounter (eg, laboratory findings). The majority of MHS beneficiaries (66% in our sample) receive both direct and purchased care. The MDR does not contain data on care provided for soldiers in combat zones or in Veterans Health Administration facilities. Before being made available for research, MDR data are thoroughly cleaned, including correction of likely coding errors, identification of data not missing at random, and imputation for missing values.^13^

### Study Population

All adults aged 18 to 64 years who received health care through the MHS between October 1, 2015, and September 30, 2018, were included in the sample, including active-duty military personnel, and their dependents, retired military personnel and their dependents, and dependent survivors. Because Medicare, rather than TRICARE, becomes the primary payer for adults at 65 years of age, we excluded beneficiaries aged 65 years and older. In addition, we excluded inactive guard/reserve and active guard/reserve (if not active duty) personnel and their dependents, due to infrequent TRICARE use.

### Variables of Interest

CKD, the primary outcome of interest, was defined by the presence of an *International Classification of Diseases, Tenth Revision* code for CKD (Table S1) and/or laboratory indicators of CKD delineated by the National Institute of Diabetes and Digestive and Kidney Diseases CKD e-phenotype, which defines CKD by estimated glomerular filtration rate (eGFR), urine albumin-to-creatinine ratio, urine protein-to-creatinine ratio, and/or dipstick urine albumin with evidence of chronicity.^14^ In this analysis, we used the more specific, less sensitive e-phenotype version to err on the side of capturing more severe, rather than less severe, CKD. The specific definition of the e-phenotype entails a urine albumin cutoff of 1+ or greater, a urinary protein-creatinine ratio cutoff of 150 mg/g or greater, and using the Black race–correcting coefficient when the race is unknown.^14^ (Using the Black race coefficient when race is unknown yields a more specific, less sensitive definition of CKD.) Beneficiaries lacking CKD laboratory results (ie, no eGFR, serum creatinine, urinary albumin-creatinine ratio, urinary protein-creatinine ratio, or dipstick urine albumin) were categorized as phenotype negative. Notably, laboratory results acquired through purchased care interactions are not available in the MDR, and thus cannot be assessed by the e-phenotype.

Primary explanatory variables included race, sponsor’s rank (a common proxy for socioeconomic status [SES] and social class),^15^^,^^16^ median household income by zip code, and marital status. For each beneficiary, race, marital status, sponsor’s military rank, and home zip code were recorded, as available, from the MDR. The sponsor’s military rank was categorized as senior officer (O-5 to O-10; WO-1 to WO4), junior officer (O-1 to O-4), senior enlisted (E-5 to E-9), or junior enlisted (E-1 to E-4). The sponsor’s home zip code was mapped to US Census Bureau data for median household income, where available, and categorized into quintiles of zip code–level median household income corresponding to areas with very low, low, medium, high, and very high incomes. Individuals missing zip codes and those from zip codes lacking median household income data were categorized as missing median household income data.

Other variables of interest included potential confounders and mediators of the association between the explanatory variables and CKD. The date of birth (to calculate age), sex, and benefits category (active duty, retired, active-duty dependent, or other dependent [retired dependents, dependent survivors]) were recorded from the MDR for each beneficiary. The sponsor’s branch of service was also captured and categorized as the Army, Air Force, Coast Guard, Marine Corps, Navy, other, or unknown. Transplant recipients and dialysis patients were defined by the presence of relevant *International Classification of Diseases, Tenth Revision* and/or Current Procedural Terminology codes (Tables S2-S5). *International Classification of Diseases, Tenth Revision* codes using value sets authored by the National Committee for Quality Assurance and published in the National Library of Medicine’s Value Set Authority Center were used to identify cases of diabetes, hypertension, depression, and HIV (Tables S6-S9). Height and weight were used to calculate BMI, with biologically implausible values for height and weight excluded (height <111.8 cm [<44 inches] or >228.6 cm [>90 inches] and weight <24.9 kg [<55 pounds] or >453.6 kg [>1,000 pounds]).^17^

### Data Analysis

Crude odds ratios (ORs) were calculated for the presence versus absence of CKD using univariable logistic regression models for each of the potential explanatory variables: race, rank, area median household income, and marital status. Age, sex, benefits category, branch of service, and depression were identified as likely confounders, as they have known or hypothesized associations with both the explanatory variables and CKD but are not likely on the pathway from social risks to CKD. Hypertension, diabetes, BMI (for overweight/obesity), and HIV were identified as likely mediators, as social risks contribute to the burden of these conditions and, in turn, these conditions increase the risk of CKD.^18^, ^19^, ^20^, ^21^, ^22^ Adjusted ORs were calculated for CKD using a series of multivariable logistic regression models: model 1 controlled for potential confounders (age, sex, benefits category, branch of service, and depression) and model 2 controlled for potential confounders and potential mediators (hypertension, diabetes, BMI, and HIV). Given the known challenges of using goodness of fit tests for logistic regression on large populations,^23^ we ran the Hosmer-Lemeshow test, as well as logistic regression covariate pattern diagnostics (ie, residuals, influence measures, delta χ^2^, “Cook’s Distance”), on a random subsample of 10,000 individuals, showing good fit. We conducted 2 sets of sensitivity analyses, first excluding individuals with missing race and/or missing median household income data, then excluding individuals who did not have at least one kidney-relevant laboratory result. Analyses were conducted using SAS, Version 9.4 (SAS Institute). This study was found exempt by the Uniformed Services University of the Health Sciences Institutional Review Board.

## Results

The total study population included 3,330,893 MHS beneficiaries, with a mean age of 33 years and a mean BMI of 28 kg/m^2^ (Table 1). The total population included 55% White (n = 1,827,435), 15% Black (n = 493,390), 10% other race (n = 314,683), 5% Asian American and Pacific Islander (n = 149,828), and 0.6% American Indian and Alaska Native (n = 21,461) beneficiaries. However, 16% of the population (n = 524,096) had missing or unknown race data. Just over half of the population (n = 1,744,766; 52%) was active duty, whereas 36% (n = 1,181.650) were dependents and 12% (n = 404,477) were retired. The majority of the population (n = 1,640,040; 49%) was senior enlisted, followed by junior enlisted at 31% (n = 1,045,845), senior officer at 10% (n = 346,183), and junior officer at 9% (n = 297,859). Hypertension, depression, and diabetes were relatively common in the population at 13% (n = 431,560), 6% (n = 207,615), and 5% (n = 151,000), respectively; whereas HIV, dialysis, and transplant were extremely rare. The area median household income was relatively high, with a median zip code–level household income of $58,121 and an interquartile range between $48,377 and $73,966. Less than half of the total population (n = 1,562,840, 47%) had a kidney test result (eGFR, serum creatinine, dipstick urine albumin, urinary albumin-creatinine ratio, or urinary protein-creatinine ratio) recorded in the MDR.

**Table 1:** Characteristics of the MHS Population With and Without CKD

Characteristics	Total	Any CKD	No CKD
n (%)	3,330,893 (100)	105,504 (3.2)	3,225,389 (96.8)
Age, y			
mean (SD)	33.0 (13.1)	47.5 (12.9)	32.5 (12.8)
median (IQR)	29 (22-42)	51 (39-58)	29 (22-41)
Female, n (%)	1,368,497 (41.1)	50,867 (48.2)	1,317,630 (40.9)
Beneficiary category, n (%)			
Active-duty Dependent	683,169 (20.5)	14,513 (13.8)	668,656 (20.7)
Retired	404,477 (12.1)	41,044 (38.9)	363,433 (11.3)
Other dependent	498,481 (15.0)	31,616 (30.0)	466,865 (14.5)
Active duty	1,744,766 (52.4)	18,331 (17.4)	1,726,435 (53.5)
Race, n (%)			
White	1,827,435 (54.9)	49,697 (47.1)	1,777,738 (55.1)
Black	493,390 (14.8)	24,551 (23.3)	468,839 (14.5)
AAPI	149,828 (4.5)	4,790 (4.5)	145,038 (4.5)
AIAN	21,461 (0.6)	372 (0.4)	21,089 (0.7)
Other	314,683 (9.5)	13,171 (12.5)	301,512 (9.4)
Unknown	156,573 (4.7)	2,996 (2.8)	153,577 (4.8)
Missing	367,523 (11.0)	9,927 (9.4)	357,596 (11.1)
Rank, n (%)			
Junior enlisted	1,045,845 (31.4)	7,952 (7.5)	1,037,893 (32.2)
Senior enlisted	1,640,040 (49.2)	79,506 (75.4)	1,560,534 (48.4)
Junior officer	297,859 (8.9)	5,333 (5.1)	292,526 (9.1)
Senior officer	346,183 (10.4)	12,712 (12.1)	333,471 (10.3)
Married, n (%)	1,675,405 (50.3)	74,393 (70.5)	1,601,012 (49.6)
Branch of Service, n (%)			
Army	1,300,105 (39.0)	39,988 (37.9)	1,260,117 (39.1)
Air Force	850,540 (25.5)	26,094 (24.7)	824,446 (25.6)
Marine Corps	383,656 (11.5)	4,317 (4.1)	379,339 (11.8)
Navy	732,309 (22.0)	33,465 (31.7)	698,844 (21.7)
Other	64,283 (1.9)	1,640 (1.6)	62,643 (1.9)
Diabetes, n (%)	151,000 (4.5)	32,503 (30.8)	118,497 (3.7)
Hypertension, n (%)	431,560 (13.0)	60,955 (57.8)	370,605 (11.5)
Depression, n (%)	207,615 (6.2)	12,362 (11.7)	195,253 (6.1)
HIV, n (%)	3,904 (0.1)	465 (0.4)	3,439 (0.1)
Dialysis, n (%)	1,772 (0.1)	1,772 (1.7)	0 (0.0)
Transplant, n (%)	1,065 (0.0)	1,065 (1.0)	0 (0.0)
BMI, kg/m^2^missing = 292,246			
mean (SD)	27.5 (5.1)	30.5 (6.1)	27.4 (5.0)
median (IQR)	26.8 (24.0-31.1)	29.4 (26.4-33.9)	26.7 (24.0-30.0)
Zip code MHI missing = 427,864			
mean (SD)	$63,789 ($22,216)	$60,145 ($19,089)	$63,922 ($22,310)
median (IQR)	$58,121 ($48,377-$73,966)	$55251 ($47,737-$67,344)	$58,237 ($48,377-$74,002)
Any proteinuria, n (%)	270,411 (8.1)	52,682 (49.9)	217,729 (6.8)
eGFR, n (%)	1,559,984 (46.8)	104,602 (99.2)	1,455,382 (45.1)
Any kidney test, n (%)	1,562,840 (46.9)	104,644 (99.2)	1,458,196 (45.2)

AbbreviationsAAPI, Asian American and Pacific Islander; AIAN, American Indian and Alaska Native; BMI, body mass index; CKD, chronic kidney disease; eGFR, estimated glomerular filtration rate; HIV, human immunodeficiency virus; IQR, interquartile range; MHI, median household income; MHS, Military Health System; SD, standard deviation.

Of the total population, 105,504 people (3.2%) had CKD identified by *International Classification of Diseases, Tenth Revision* codes and/or laboratory values indicative of CKD (Table 1). Compared to beneficiaries without CKD, those with CKD were on average older, less likely to be active duty, more likely to be retired, more likely to be Black, more likely to be senior enlisted or a senior officer, and more likely to be married. Beneficiaries with CKD also had a higher average BMI and were more likely to have hypertension, diabetes, and depression compared to those without CKD. Almost all individuals with CKD had at least one measure of eGFR recorded in the MDR (n = 104,602; 99%), but only half (n = 52,682; 50%) had a measure of proteinuria.

Table 2 provides crude, confounder-adjusted, and confounder-mediator-adjusted ORs for the associations between sociodemographic factors and CKD. In crude analyses, both Asian American and Pacific Islander (OR, 1.18; 95% confidence interval [CI], 1.15-1.22) and Black (OR, 1.87; 95% CI, 1.84-1.90) beneficiaries had elevated prevalence of CKD compared to White beneficiaries; however, statistical significance was lost for Asian American and Pacific Islander beneficiaries after adjusting for confounders. In confounder-adjusted models, we found that Black beneficiaries had 1.67 times higher odds of prevalent CKD compared to their White counterparts. As expected, when additionally adjusting for suspected mediators, the association between Black race and CKD was partially but not completely mitigated (OR, 1.30; 95% CI, 1.28-1.32).

**Table 2:** Crude, Confounder, and Confounder-Mediator-Adjusted Associations Between Sociodemographic Factors and CKD in the Adult MHS Population, October 1, 2015, Through September 30, 2018

Variable	Effect	Crude OR (95% CI)	Confounder-Adjusted OR (95% CI)	Confounder & Mediator-Adjusted OR (95% CI)
Race	White	1.0	1.0	1.0
	AAPI	1.18^a^ (1.15-1.22)	0.98 (0.95-1.01)	0.87^a^ (0.84-0.90)
	Black	1.87^a^ (1.84-1.90)	1.67^a^ (1.64-1.70)	1.30^a^ (1.28-1.32)
	AIAN	0.63^a^ (0.57-0.70)	0.94 (0.84-1.04)	0.88^a^ (0.79-0.98)
	Other	1.56^a^ (1.53-1.59)	1.30^a^ (1.27-1.32)	1.17^a^ (1.15-1.20)
	Unknown	0.70^a^ (0.67-0.72)	0.73^a^ (0.70-0.76)	0.77^a^ (0.74-0.80)
Rank	Senior officer	1.0	1.0	1.0
	Junior officer	0.48^a^ (0.46-0.49)	1.09^a^ (1.06-1.13)	1.00 (0.97-1.03)
	Senior enlisted	1.34^a^ (1.31-1.36)	1.73^a^ (1.69-1.76)	1.33^a^ (1.30-1.35)
	Junior enlisted	0.20^a^ (0.20-0.21)	1.32^a^ (1.28-1.37)	1.02 (0.99-1.06)
Marital	Married	1.0	1.0	1.0
Status	Single	0.41^a^ (0.41-0.42)	0.77^a^ (0.76-0.79)	0.82^a^ (0.81-0.83)
Income n = 427,864 missing	Very high quintile (>$78,729)	1.0	1.0	1.0
	High quintile ($62,959-$78,729)	1.23^a^ (1.20-1.26)	1.40^a^ (1.36-1.44)	1.32^a^ (1.29-1.35)
	Middle quintile ($53,440-$62,958)	1.54^a^ (1.51-1.57)	1.98^a^ (1.94-2.02)	1.85^a^ (1.81-1.89)
	Low quintile ($46,366-$53,439)	1.74^a^ (1.71-1.78)	2.76^a^ (2.70-2.82)	2.59^a^ (2.53-2.64)
	Very low quintile (<$46,366)	1.43^a^ (1.40-1.46)	2.58^a^ (2.52-2.64)	2.38^a^ (2.32-2.43)
	Missing	0.29^a^ (0.28-0.30)	0.92^a^ (0.89-0.96)	0.91^a^ (0.87-0.95)

AbbreviationsAAPI, Asian American and Pacific Islander; AIAN, American Indian and Alaska Native; CI, confidence interval; CKD, chronic kidney disease; MHS, Military Health System; OR, odds ratio.

a Statistical significance.

Compared to senior officers, senior enlisted beneficiaries had higher odds of CKD in crude analyses (OR, 1.34; 95% CI, 1.31-1.36), whereas junior officers (OR, 0.48; 95% CI, 0.46-0.49) and junior enlisted (OR, 0.20; 95% CI, 0.20-0.21) beneficiaries had lower odds of CKD. However, after adjusting for suspected confounders, all ranks below senior officer had elevated odds of prevalent CKD. After further adjusting for suspected mediators, statistical significance was lost for both junior officer and junior enlisted beneficiaries, whereas the odds of CKD remained statistically significant for senior enlisted beneficiaries (OR, 1.33; 95% CI, 1.30-1.35).

Compared to married beneficiaries, those who were single had lower odds of CKD in crude and adjusted analyses; however, the magnitude of the protective effect of single status dropped from an OR of 0.41 (95% CI, 0.41-0.42) in the crude analysis to 0.77 (95% CI, 0.76-0.79) in the confounder-adjusted model and 0.82 (95% CI, 0.81-0.83) in the confounder- and mediator-adjusted models.

In crude, confounder-adjusted, and mediator-adjusted models, a decreasing zip code–level median household income was associated with increasing odds of prevalent CKD through all quintiles except the lowest. Of note, 13% of the total sample was missing median household income data. After adjusting for confounders, the magnitude of the association increased across all median household income quintiles. Compared to the very high median household income quintile, the high quintile had 1.40 (95% CI, 1.36-1.44) times greater odds of CKD, the medium quintile had 1.98 (95% CI, 1.94-2.02) times greater odds of CKD, the low quintile had 2.76 (95% CI, 2.70-2.82) times greater odds of CKD, and the very low quintile had 2.58 (95% CI, 2.52-2.64) times greater odds of CKD. After further adjusting for suspected mediators, the magnitude of the association was attenuated but remained significant for all median household income levels. In sensitivity analyses, the overall pattern of increased prevalence of CKD among Black beneficiaries, beneficiaries of lower rank, and beneficiaries living in lower-income areas remained consistent (Tables S10-S13).

## Discussion

This analysis found that racial and socioeconomic disparities exist in the MHS despite universal health care coverage. In confounder-adjusted models, Black MHS beneficiaries had 1.67 times higher odds of prevalent CKD compared to their White counterparts, which is consistent with the elevated odds of CKD among Black Americans recently reported by the United States Renal Data System.^18^ Individuals of lower compared to higher SES and social class in the MHS experienced higher prevalence of CKD, with ORs ranging from 1.32 (junior enlisted compared to senior officer) to 2.76 (fourth compared to first quintile of median household income). These increased odds of CKD are consistent with a recent meta-analysis of US studies, which found a pooled OR of prevalent CKD in low-income compared to high-income groups of 1.55.^24^

Genetic differences, such as the elevated prevalence of high-risk *APOL1* risk variants in Black Americans, may partially account for differences in CKD prevalence by race found within the MHS.^8^ However, race is increasingly recognized as a poor proxy for underlying genetics.^25^^,^^26^ Whereas certain genetic variants, such as *APOL1*, are associated with Black race, the social construct of race does not accurately reflect or completely align with the genetic differences resulting from ancestral origin.^26^ Among Americans who identify as Black, roughly one-quarter of ancestry informative genetic markers suggest non-African origin, likely in part as a result of European colonization and the forced enslavement of Africans in America.^27^^,^^28^ Instead, race reflects a complex mixture of social, cultural, and biological factors.^25^

The presence of SES/social class disparities in CKD within the MHS supports a role for social factors in Black-White CKD disparities. Indeed, structural racism and social determinants of health are inextricably linked, as structural racism mediates access to wealth and material resources for marginalized race groups and underlies the inequitable distribution of the social determinants of health. The role of low SES in racial disparities in kidney mortality has been demonstrated in analyses of merged United States Renal Data System Census data for 11,027 end-stage renal disease patients, which found elevated mortality rates for Black compared to White end-stage renal disease patients were attenuated in high- versus low-SES neighborhoods after adjusting for baseline demographics, clinical characteristics, rurality, and access to care.^29^ Similarly, in participants of the Americans' Changing Lives study followed over 25 years, adjusting for SES fully attenuated the increased risk of death in Black compared to White individuals with CKD.^30^

The presence of SES/social class disparities in CKD within the MHS further suggests that the presence of health care coverage alone is not sufficient to mitigate disparities. Health care coverage does not equate to equitable delivery of care, and thus disparities in care delivery may be present within the MHS and contribute to both racial and SES/social class disparities in CKD. Prior research in the MHS has shown that some care delivery disparities are present for some^6^^,^^31^^,^^32^ but not other^33^^,^^34^ care pathways in the MHS. In addition, social risks that are disproportionately prevalent in marginalized populations may contribute to both racial and SES disparities in CKD found in the MHS. Social risks, such as lack of transportation, lack of childcare, and competing time and resource priorities, may impede access to health care services despite universal health care coverage.^35^^,^^36^ Among publicly insured adults enrolled in Minnesota Health Care Programs, reported barriers to care included an inability to cover out-of-pocket costs, transportation limitations, clinic hours that conflicted with other responsibilities, and lack of childcare.^36^ A population of majority low-income, African American “safety net” CKD patients reported similar barriers despite insurance coverage, including transportation difficulties, financial challenges, and a lack of work leaves.^35^

Social risks may also contribute to the racial and SES disparities in CKD seen in the MHS through numerous pathways outside of the health care system. These include increased stress and allostatic loads, increased risk behaviors (eg, smoking), barriers to health-promoting behaviors, reduced health literacy and knowledge, reduced social support, and an increased risk of environmental exposures.^1^ However, data are limited to assess the relevance of these pathways in the MHS. Enlisted rank, high psychosocial stress, and low levels of social support were each associated with an increased prevalence of overweight/obesity in Army spouses.^37^ Smoking is more common among enlisted military personnel compared to officers,^38^ suggesting that this risk behavior could contribute to an increased CKD risk among lower-rank individuals.

Our finding that single beneficiaries had lower odds of CKD compared to married beneficiaries was counter to our initial hypothesis, that being married, as a form of social support, would reduce the risk of CKD. Instead, this finding may reflect ascertainment bias, as married individuals may be more likely than their single counterparts to access care (and thus laboratory results) due to encouragement from their spouse. Alternatively, being married in the MHS may represent a source of stress, at least among the active-duty population, as one spouse may be frequently deployed.

Our findings are consistent with prior findings relating to both racial and SES/social class disparities in CKD in populations with universal health care coverage. Studies in countries that provide universal health care coverage, including the United Kingdom, Denmark, and Australia, have found that low SES is associated with an increased CKD prevalence, an elevated end-stage renal disease incidence, and reduced dialysis survival.^9^, ^10^, ^11^ A systematic review of 25 studies assessing the racial disparities in mortality within the Veterans Health Administration found that mortality among Black beneficiaries was similar to or lower than that among White beneficiaries; however, when narrowed to individuals with CKD, mortality rates in Black compared to White beneficiaries were modestly elevated.^7^ Further, in a sample of 56,767 veterans with stage 3 or 4 CKD, Black veterans were more likely than their White counterparts to progress to end-stage renal disease despite universal access to care and higher rates of nephrology referral for Black compared to White veterans.^8^

Of note, the MHS population has several unique aspects that distinguish it from the general US population, which may limit the generalizability of our findings. Since access to the MHS system is achieved through employment in (or retirement from) the military, the MHS population may be more economically stable than the general US population. In addition, military service members often have access to benefits that are not typically available in the general US population, such as subsidized childcare, savings on food expenses through commissaries, and educational support. However, these subsidies are not universally available, they do not address all social risks, and their health benefits may be countered by the negative health effects of increased stress and demands associated with individual and family military service (eg, deployment, combat, separations).

This analysis is among the first studies assessing the burden of CKD in the MHS and provides additional context for understanding the role of universal health care coverage in racial and SES/social class disparities in CKD. Strengths of the study include the large sample size and the use of a validated, laboratory value-based e-phenotype—in combination with diagnosis codes—to improve the sensitivity of CKD detection. However, we must acknowledge important limitations. The administrative data used in this analysis are intended for use in claims adjudication and not research, and thus have inherent shortcomings. Because the data are cross-sectional, causal links between race or SES/social class and CKD prevalence cannot be inferred. Data are available on only a small subset of social determinants in the MHS, and thus we cannot account for the role of many relevant social factors in the associations between SES, race, and CKD prevalence. Because we lack laboratory data for purchased care interactions, our analysis may have missed laboratory results indicative of CKD, and thus we may have underestimated the true prevalence of CKD. However, few health care systems are closed, and the exchange of data across health systems is poor; thus, any analysis of health care data will suffer from such absence of data. Particularly, if purchased and direct-care use varies by race or SES, such misclassification may introduce bias. In addition, because the Black race–correcting coefficient was used in calculating the eGFR for those with unknown race (to provide a more specific measure of CKD), eGFRs in this population may be inflated, and we may underestimate the prevalence of CKD. Given the recently identified disparity in CKD among Pacific Islanders,^18^ the combined Asian American and Pacific Islander race category available from the MDR may mask CKD disparities in the MHS Pacific Islander population. Whereas rank is commonly used to represent SES,^15^^,^^16^ it does not perfectly align with the 3 traditional components of SES (income, education, and occupation) and may also reflect differences in social standing in the context of the military hierarchy, as well as differences in health knowledge, attitudes, and beliefs, which may influence health and care-seeking behaviors, as well as the quality of care and, ultimately, CKD outcomes. The transient nature of the MHS population may limit the accuracy and usefulness of the zip code–level median household income data, as a beneficiary’s most recent zip code may not accurately reflect long-term exposure to area deprivation.

Despite the universal health care coverage provided through the MHS, racial and socioeconomic CKD disparities exist in this population. Our findings are consistent with racial and socioeconomic CKD disparities identified in other domestic and international settings that provide universal health care coverage. Genetic differences may partially account for the racial differences in CKD in insured populations. However, the existence of disparities by rank and zip code–level median household income suggests that SES, social class, and associated social risks may increase the risk for CKD despite access to universal health care coverage. Therefore, access to health care coverage alone may not be sufficient, and broader interventions to address social risk factors may be necessary to significantly mitigate racial and socioeconomic CKD disparities.
